# More Prevalent Prescription of Medicine for Hypertension and Metabolic Syndrome in Males from Couples Undergoing Intracytoplasmic Sperm Injection

**DOI:** 10.1038/s41598-018-32813-4

**Published:** 2018-09-28

**Authors:** A. Elenkov, Y. Al-Jebari, A. Giwercman

**Affiliations:** 1 Skåne University Hospital Malmö, Reproductive Medicine Center, Malmö, Sweden; 2 Lund University, Dept. of Translational Medicine, Molecular Genetic Reproductive Medicine, Malmö, Sweden; 3 Medical University Sofia, Dept. of Urology, Sofia, Bulgaria

## Abstract

Register-based studies have indicated that men with impaired fertility are at higher risk for developing various adult-onset diseases than fertile men. The majority of men undergoing ICSI treatment are sub-fertile and since they are in contact with the health care system, these men are well suited as target for preventive measures. Our study included all men (N = 459 766) who had fathered children in Sweden between 2006 and 2016. Swedish registry data was used for obtaining information regarding conception method and defining three groups of fathers – ICSI -treated, IVF – treated and non IVF/ICSI. By sourcing data from the Swedish Prescribed Drug Register, we specifically searched for information regarding prescription and usage of at least one prescription for diabetes mellitus, hypertension (HT) or dyslipidemia to serve as a proxy for metabolic disease among the study groups. If all three types of medicine were prescribed, the patient was considered as having metabolic syndrome. Our results indicate male partners in couples who became parents using ICSI to be at higher risk for being treated for hypertension (HR = 1.15 95% CI: 1.06–1.24, p = 0.001) and metabolic syndrome (HR = 1.28 95% CI: 1.01–1.58, p = 0.042) when compared to non IVF/ICSI men.

## Introduction

There is an increasing focus on association between male infertility and risk of chronic nonmalignant diseases^1^. A number of studies have established a link between reduced male fecundity and morbidities including cardiovascular, pulmonary, renal diseases as well as dementia and diabetes mellitus^2–4^. These findings suggest that the status of male fertility and semen quality can potentially be regarded as universal health markers^5^. Animal and human studies show up to 15% of the genome to be directly involved in the reproduction^6^. In this context regulators of non-reproductive, including metabolic, pathways are likely to have an impact on reproductive function and vice versa. It has also been suggested that a combination of genetic factors, lifestyle or environmental exposures, starting as early as in human prenatal life, can cause impairment of male reproductive function as well as other adult–onset diseases^7,8^.

Metabolic syndrome (MetS) is defined as a cluster of metabolic disorders. Different definitions have been proposed, all of them including glucose intolerance (high fasting glucose blood level), dyslipidemia (high triglycerides or low levels of high-density lipoprotein cholesterol blood levels), hypertension and obesity^9,10^. MetS might be related to spermatogenesis and sexual function through different pathways. Biochemical signs of hypogonadism have been found in 30% of men with fertility problems and impaired semen quality. Testosterone deficiency is a well-known marker of increased risk of metabolic and cardiovascular disease as well as premature mortality^4,11–13^.

Previous research has pointed to the fact that men with impaired fertility are overrepresented among fathers to children conceived by intracytoplasmic sperm injection (ICSI) and thus it can serve as feasible proxy for male infertility in large population based cohorts^7^.

We aimed to investigate whether male partners from couples undergoing ICSI treatment, had an increased prevalence of prescription of medicine for different components of MetS. IVF couples are suggested not to be representative for the entire population due to the lower morbidity in connection to higher socio economic status^14,15^. Thus we decided to compare ICSI treated men to those who conceived without use of these assisted reproduction techniques (ART, addressed as control group from now on) by using Swedish population based register data.

## Materials and Methods

### Study population

In Sweden all inhabitants have a unique 10-digit personal number. In order to preserve anonymity, all cross-linking between the registries were done by the Swedish National Board of Health and Welfare and, subsequently, before the data were given to the researchers, the 10-digit personal number was replaced by a code not allowing identification of study subjects. The study has been approved by the Regional Ethical Board in Lund (No: 2015/670).

The search base for our cohort were all children registered in the Medical Birth Register (MBR) born alive in Sweden after the start of the Swedish prescription drug register (SPDR) during the period January 1st 2006–September 1st 2016 and their mothers. MBR contains information on 97–99% of all births in Sweden from 1973 onwards (Socialstyrelsen, 2010). By cross-linking the codes of the mothers to Swedish Total Population Register and the Swedish Multigenerational Register we identified the fathers. MBR contains data including the birth date and the gestational week of the newborn. This information allowed us to calculate the approximate time of conception. Cases of missing paternal serial number, missing conception estimate for child (because of missing gestational age at birth) were regarded as missing data and were excluded from our analysis.

### Information on fertility status

By assuming that infertile men are overrepresented among fathers to children conceived with ICSI we decided to use Swedish register data to distinguish three groups of fathers: ICSI – treated, IVF – treated and non-IVF/ICSI (control) group. To avoid bias introduced by fathers being counted multiple times, only the conception method for the first child born within our cohort was used for categorising the father into one of the three groups.

**Figure 1:**
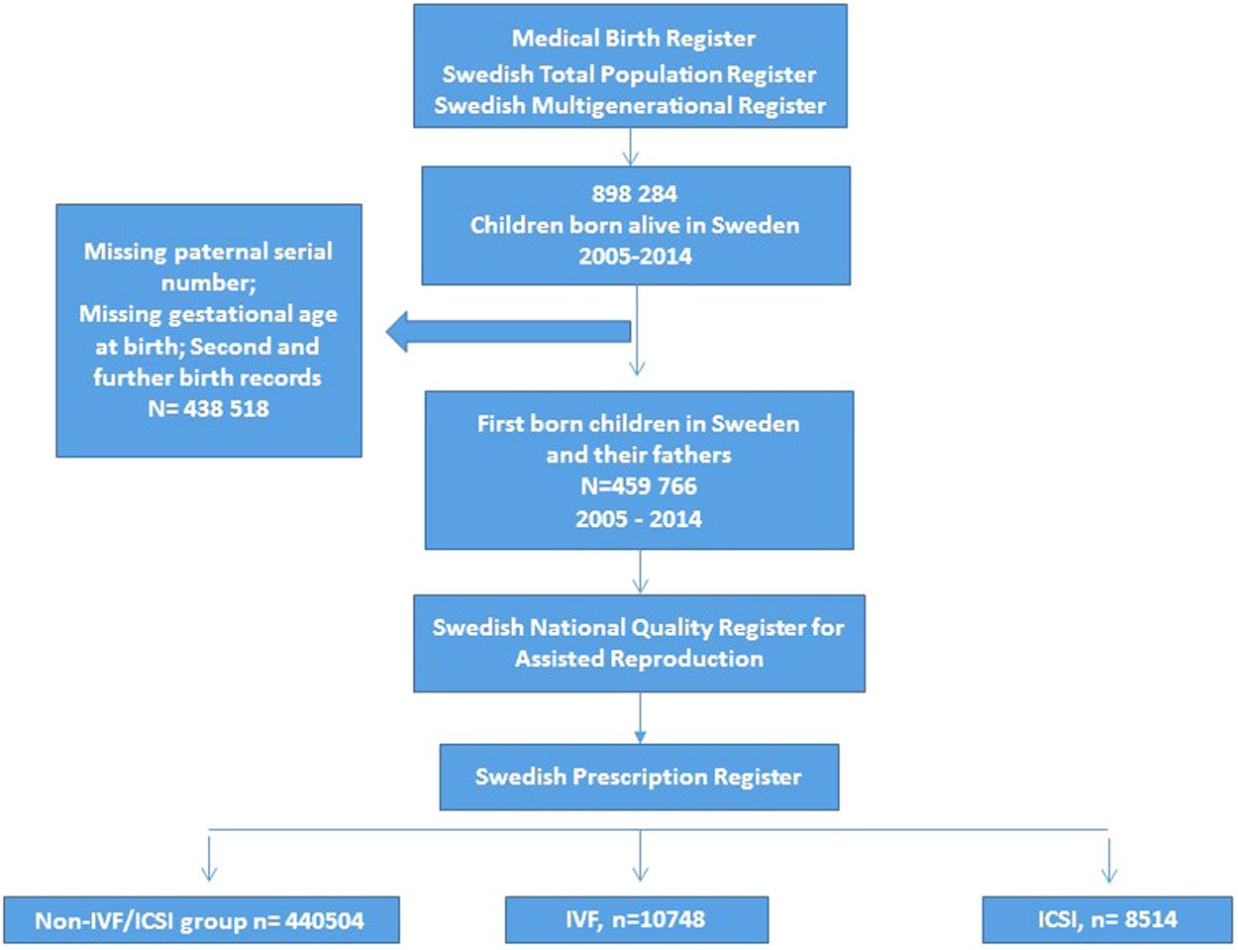
Data acquisition using Swedish population based registries.

Subsequently we matched the data on mothers and fathers with the Swedish National Quality Register for Assisted Reproduction (Q-IVF). It contains information on assisted reproduction conception method (ICSI or IVF) for all births in Sweden from 2007 and onwards. Information regarding the conception method before 2007 was gathered from the MBR. The men were then categorized in three groups – ICSI, IVF and non IVF/ICSI (control group) - according to the mode of conception for the first child registered in MBR (Fig. 1).

### Defining subgroups of fathers with disease

We used the SPDR to look at pattern of filled drug prescriptions for diabetes mellitus (DM), hypertension (HT) and dyslipidemia (DLE). Corresponding generic medications of at least one prescription for each disease were used as proxy to identify men treated for DM, HT, or DLE among the study groups. By using the Adult Treatment Panel III (ATPIII) criteria we used three filled prescriptions (one for DM, HT and DLE) for one man to serve as proxy for MetS^9^.

SPDR spans from 1 July 2005 onwards covering all drug prescriptions filled using pharmaceutical (generic names). More than 99% of all prescriptions are estimated tо be reported in SPDR^16^. We specifically searched for information regarding prescription of medicines for HT, DM and DLE. The list of medications identified in SPDR is summarized in Table 1.

**Table 1:** Generic names of medications and corresponding therapeutic groups, identified through the Swedish prescription drug register (SPDR).

Antidiabetic Medications	Antihypertensive and Cardiovascular Medications	Lipid Lowering Medications
Akarbose Exenatide Glibenklamide Gliklazide Glimepiride Glipizide Insulin Klorpropamide Liraglutide Metformin Nateglinide Pioglitazon Repaglinide Rosiglitazon Sita-gliptine Tolazamide Tolbutamide Vildagliptine.	Adenosine Ajmaline Aliskirene Alprostadil Amilorid Amiodaron Amrinon Angiotensin-amid Benazepril Cilazapril Digitoxin Digoxin Diltiazem Disopyramin Dobutamine Dopamine Dronedarone Ena-lapril Eplerenone Eprosartan Etilefrin Fosinopril Glyceryltrinitrate Ibutilide Indometacine Irbesartan Isoprenaline Iva-bradine Levosimendane Lisinopril Lo-sartan Mexiletin Midodrin Milrinone Moexipril Molsidomine Olmesartan-medoxomil Perindopril Prenalterol Pro-pafenone Ramipril Regadenosone Telmi-sartan Tolvaptan Trandolapril Triamteren Trimetazidin Valsartan Verapamil Verna-kalant.	Acipimox Atorvastatin Bezafibrat Cerivastatin Ezetimibe Fenofibrat Fluvastatine Gemfibrozil Lomitapid Lovastatin Niceritrol Pravastatin Rosuvastatin Simvastatin.

### Statistical analysis

Assuming common etiology behind MetS or its components and reduced male fertility possibly arising early in life, the follow-up period, for the cohort, was defined as starting at the time of the birth of the father and continuing until the earliest day of registration of prescription of medication in SPDR or end of follow-up.

In order to evaluate the post-infertility treatment risk of prescription, we performed a sub-analysis. The follow-up time was defined as starting from the time of child of conception to a given father continuing until the earliest registration of prescription or end of follow up. We excluded all fathers from our cohort who had filled drug prescriptions before their child was conceived.

Another analysis was performed in order to evaluate the risk of prescription medicines before the child conception. The follow up time was defined as starting from the birth of the father, continuing until the earliest date of prescription or the conception of the first child. All fathers who have had prescription medicines after conception were excluded from analysis.

Hazard ratios (HR) of filled prescriptions for DM, HT and DLE in patients treated for ICSI and IVF with corresponding confidence intervals (CI) were calculated using Cox proportional hazards models using the non IVF/ICSI treated male population as reference. In the Cox regression model age was corrected for as follows:

1. Using the age of the father as underlying time variable, when evaluating the risk from father’s birth;
2. Included as co-variate when evaluating – in the sub-analysis - the post conception risk only.

Data on paternal educational level was sourced from the Swedish Educational Register and was used to correct for socioeconomic status by using paternal years of formal education as an indicator (3 categories −10 years or less; 10–14; 15 or more years or missing data). It was used as a co-variate in the Cox regression model. All analyses were conducted in SPSS version 24.0.0.1 (IBM Corp, Armonk, NY) and Python version 3.6.1. (Python Software Foundation, python.org). All statistical analyses were two-sided and p < 0.05 was considered statistically significant.

## Results

### Description of the cohort

A total of 898 284 children were born alive in Sweden during the study period. After excluding the cases with missing data along with second and further birth records, linked to the same father, we ended up with of 459 766 first-born children and their fathers. A total of 8514 men were treated with ICSI (1.9%), 10748 with IVF (2.3%) and 440504 made the control (non-IVF/ICSI) group.

The mean age of the fathers at the time of the first prescription of medicine was as follows: 39.2 years for the ICSI group, 39.1 years for the IVF group and 37.1 years for the control group. The age of conceiving the first child was 35.9 years for the ICSI, 35.4 for the IVF and 31.1 for the non-IVF/ICSI group.

### ICSI fathers

**Figure 2:**
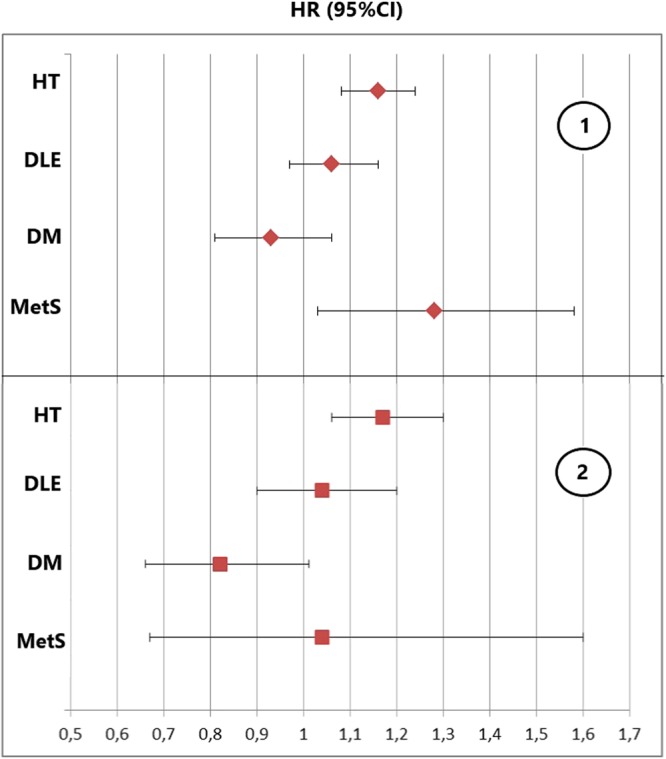
Hazard ratios (HR) and 95% confidence intervals for prescription medicines for diabetes mellitus (DM), hypertension (HT), dyslipidemia (DLE) and metabolic syndrome (MetS) among males undergoing ICSI when compared to the control group: 1: Without exclusion for medications before the estimated time of conception, with follow up time from father birth (after adjustment for educational level); 2: Analysis evaluating the post infertility treatment risk by excluding fathers on medication prior to child conception and following them from child conception date (after adjustment for age and educational level).

ICSI-treated men were found to be with higher risk to be prescribed for medicines for HT (HR = 1.15 95% CI: 1.06–1.24, p = 0.001). Filled prescription of medications for MetS were also found to be significantly higher among the ICSI treated group (HR = 1.28 95%CI: 1.01–1.58, p = 0.042). In the ICSI treated group 0.9% of the men were treated for MetS compared to 0.4% in the control group. (Figs 2:1; Fig. 3; Tables 2 and 3).

**Figure 3:**
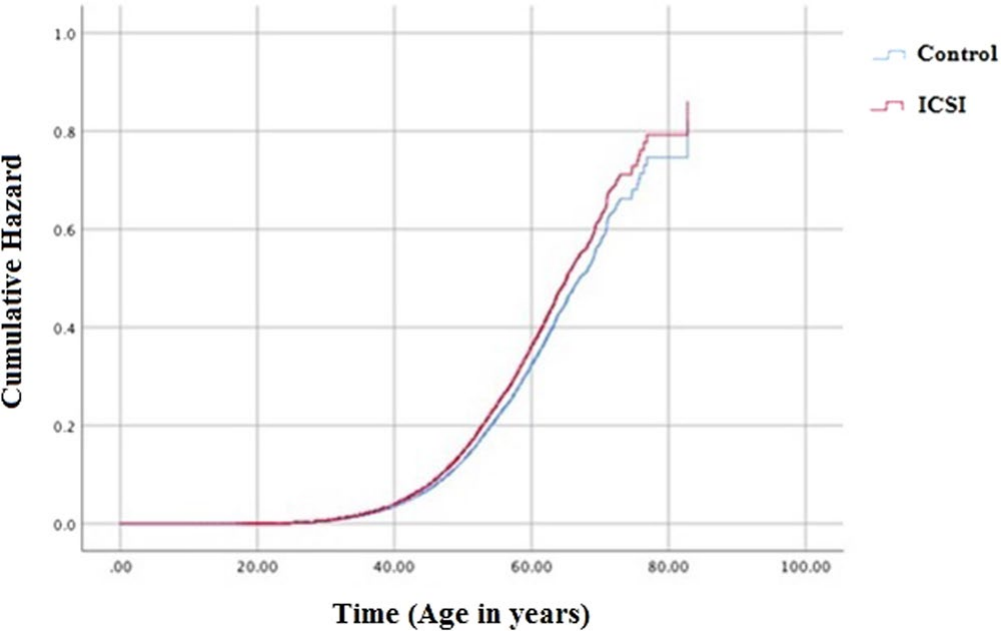
Cox regression showing the cumulative hazard of prescribing medicines for hypertension in ICSI treated men when compared to controls, followed from father birth, after adjustment for educational level.

**Table 2:** Distribution of prescription medicines for treatment of diabetes mellitus (DM), hypertension (HT), dyslipidemia (DLE) and metabolic syndrome (MetS) and corresponding hazard ratios (HR) after adjustment for educational level among men starting the follow up from father birth (after adjustment for educational level), until first prescription of medicine or end of follow up.

	ICSI (N = 8514)				Non IVF/ICSI (N = 440504)	
	%	N	HR (95% CI)	P-value	%	N
DM	2.3%	194	0,92 (0,79–1,07)	0,26	1.5%	6712
HT	8.7%	741	1.15 (1.06–1.24)	0,001	4.7%	20681
DLE	4.9%	418	1.03 (0.93–1.14)	0,61	2.8%	12180
MetS	0.9%	80	1.28 (1.01–1.58)	0,042	0.4%	1820

**Table 3:** Hazard ratios (HRs) for prescription of drugs for diabetes mellitus (DM), hypertension, (HT) dyslipidiemia (DLE) and metabolic syndrome (MetS) in men treated with IVF and ICSI – as compared to controls (non IVF/ICSI) - when followed from the time of father birth until first prescription of medicine or end of follow up.

		Univariate Cox regression		After adjustment for educational level	
		HR (95% CI)	p-value	HR (95%CI)	p-value
ICSI	DM	0.91 (0.78–1.06)	0,22	0,92 (0,79–1,07)	0,26
	HT	1.14 (1.06 – 1.23)	0,001	1.15 (1.06–1.24)	0,001
	DLE	1.03 (0.92 – 1.35)	0,64	1.03 (0.93–1.14)	0,61
	MetS	1.26 (0.99 – 1.58)	0,054	1.28 (1.01 – 1.58)	0,042
IVF	**DM**	0,69(0,59–0,81)	<0,001	0,70(0,60–0,83)	<0,001
	**HT**	0,9 (0,83–0,98)	0,013	0,92 (0,85–0,99)	0,034
	**DLE**	0,96 (0,87–1,06)	0,39	0,97 (0,87–1,07)	0,5
	**MetS**	0,78 (0,58–1,04)	0,86	0,8 (0,6–1,07)	0,13

Our sub-analysis including only post-conception initiated prescriptions showed again higher risk for being prescribed anti- HT drugs in the ICSI group (HR = 1.18; 95% CI: 1.06–1.30). The mean time between ICSI procedure and prescription of anti-HT medicine was 5.6 years and 5.8 years for the control group. Risk estimates for prescriptions for DM, DLE and MetS in the ICSI group were not statistically significantly altered in comparison to the control group (Fig. 2:2, Tables 4 and 5). When the risk for prescription medicines before child conception was evaluated, ICSI fathers were found to be with higher risk for DLE and HT (Table 6).

**Table 4:** Distribution of prescription medicines for treatment of diabetes mellitus (DM), hypertension (HT), dyslipidemia (DLE) and metabolic syndrome (MetS) with corresponding hazard ratios (HR) evaluating the post infertility treatment risk among the study groups (after adjustment for age of conception and educational level).

	ICSI				IVF				Controls (Non IVF/ICSI)		
	HR (95% CI)	P-value	%	N (Total)	HR (95% CI)	%	N (Total)	P-value	HR	%	N (Total)
DM	0.84 (0.67–1,04)	0.1	1.0	87 (7783)	0,72 (0,58–0.89)	0.9	89 (9815)	0,002	ref	0.9	3805 (399055)
HT	1.176 (1.064–1.300)	0,002	5.2	397 (7289)	0,96 (0,87–1,07)	4.1	401 (9360)	0,47	ref	3.2	12914 (396699)
DLE	1.04 (0.91–1.19)	0.56	2.8	221 (7583)	1.00 (0.89–1.14)	2.6	257 (9577)	0,91	ref	1.9	7679 (394616)
MetS	1.07 (0.98–1.17)	0.15	0.3	21 (7527)	0.65 (0.39–1.09)	0.2	15 (9630)	0,1	ref	0.2	687 (396099)

**Table 5:** Hazard ratios (HRs) for prescription of diabetes mellitus (DM), hypertension, (HT) dyslipidiemia (DLE) and metabolic syndrome (MetS) medication in men treated with IVF and ICSI – as compared to controls (non IVF/ICSI) - when followed from the time of child conception.

		Univariate cox regression		After adjustment for age of conception		After adjustment for age of conception and educational level	
		HR (95% CI)	P-value	HR (95% CI)	P-value	HR (95% CI)	P-value
ICSI	DM	1.28 (1.04–1.58)	0,023	0.82 (0.66–1.01)	0.09	0.84 (0.67–1,04)	0.1
	HT	1.73 (1.57–1.91)	<0.001	1.173 (1.062–1.297)	0,002	1.176 (1.064–1.300	0,002
	DLE	1.61 (1.41–1.84)	<0.001	1.04 (0.9–1.2)	0.57	1.04 (0.91–1.19	0.56
	MetS	1.77 (1.15–2.73)	0.01	1.04 (0.67–1.6)	0.8	1.07 (0.98–1.17)	0.15
IFV	DM	0,98 (0,79–1,21)	0,85	0,70 (0,57–0,87)	0,001	0,72 (0,58–0.89)	0,002
	HT	1,30 (1,79–1,44)	<0,001	0,95 (0,86–1,05)	0,33	0,96 (0,87–1,07)	0,47
	DLE	1,40 (1,24–1,59)	<0,001	1,00 (0,88–1,13)	0,99	1,00(0,89–1,14)	0,91
	MetS	0,92 (0,55–1,53)	0,74	0,63 (0,38–1,05)	0,08	0,65 (0,39–1,09)	0,10

### IVF fathers

When we compared the IVF-treated men to the control group we found lower risk for DM prescriptions (HR = 0,70; 95% CI: 0,60–0,83, p < 0,001) and HT (HR = 0,92; 95% CI: 0,85–0,99, p = 0,034, Table 3); and no statistically significant associations for DLE or MetS (Table 3). Similar risk reduction (HR:0,72; 95% CI: 0,58–0.89, p = 0,002), as considers prescription of anti-DM drugs, was found in the sub-analysis dealing with post-fertility treatment prescription only. No statistically significant association were established for the time before the child conception (Table 6). All estimates remained robust after adjustments for educational level (Tables 3–5).

**Table 6:** Distribution of prescription medicines for treatment of diabetes mellitus (DM), hypertension (HT), dyslipidemia (DLE) and metabolic syndrome (MetS) with corresponding hazard ratios (HR) evaluating the treatment risk before child conception for ICSI and IVF fathers. All results are adjusted for educational level.

	ICSI		IVF	
	P- value	HR (95% CI)	P value	HR (95% CI)
DM	0,153	1,16 (0,94–1,43)	0,068	0,79 (0,62–1,01)
HT	<0,001	1,37 (1,22–1,55)	0,83	1,02 (0,88–1,17)
DLE	0,002	1,28 (1,09–1,51)	0,14	1,14 (0,96–1,36)
MetS	0,067	1,55 (0,97–2.46)	0,66	1,14 (0,64–2,03)

## Discussion

Our analysis identified male partners in couples who became parents using ICSI to be at higher risk for being treated for HT and MetS with filled prescription medicines when compared to men who did not use ART to conceive their first child. Furthermore, ICSI treated men had higher risk to be prescribed to medicines for HT in the years after child conception.

These findings point to different possible mechanisms linking impairment of male fertility to the symptoms of MetS: metabolic disease or its treatment as factor contributing to infertility and/or common underlying cause of both conditions.

There is a limited amount of reports studying the association between male infertility and hypertension. By using rat model the link between renovascular hypertension, decreased sexual behaviour and impaired spermatogenesis was attributed to imbalances in FSH, testosterone and prolactin^17^. Hypertensive men were found to be with higher levels of clusterin (glycoprotein associated with abnormal sperm morphology) and sperm DNA damage^18,19^. Previous research has established a link between hypertension, cardiovascular disease and endocrine axis abnormalities^11,20–22^. Some of the drugs used to treat hypertension such as calcium–channel and beta blockers have been associated with impairment of acrosomal receptors and many other semen abnormalities^23,24^. Our analysis established strong association between filled prescriptions for HT among ICSI-treated men regardless of whether we defined the birth of the father or the time of the infertility treatment as start of the follow-up period. The latter cannot exclude both the probability of some suppressive effect of some medications for HT on male fertility^23^, or the possibility that some common underlying mechanism drives both reduced male fecundity and higher risk for cardiovascular disease.

Our analysis revealed statistically significant increase in the risk for MetS in the ICSI group when compared to the controls. Metabolic disturbances are known to be more pronounced in hypogonadal sub-fertile men which indicates the role of the testosterone levels as a potential mediator for disease^4^. The figures in our cohort, as percentage, are relatively low in contrast with the prevalence of the syndrome in other big studies where MetS is estimated to be present in 22–34% of men with fertility problems^25^. This might be caused by the fact that our methodology cannot take into account the occurrence of components of MetS which is not treated by prescribed medicine but rather by lifestyle changes. These patients are not identifiable through prescription register and probably compose the majority of the cases. In addition, our cohort consists primarily of young men, a fact which in combination with the relatively short follow up time might imply that only a minor proportion of the patients being at risk for developing metabolic syndrome has been prescribed medication. Our findings point to, probably, most severe forms of MetS being more common among ICSI fathers.

ICSI-treated fathers in our cohort showed increased risk for prescribing medicines for DLE in the time before the child is conceived. Sub-fertile men with subnormal testosterone have been shown to have higher trygliceride levels when compared to eugonadal sub-fertile men thus suggesting a possible common mechanism^4^. In the LIFE study, a correlation was found between increased total cholesterol levels and phospholipids and decreases in seminal volume, smaller sperm head size, lower percentage of sperm with intact acrosome and longer time to pregnancy^26^.

The pathogenetic mechanism behind our findings is not clear. However, previous studies have shown that children born small at gestational age are more likely, later in life to undergo ICSI treatment and also to develop various metabolic disorders^7,27^. This would point to prenatal factors as common cause of sub fertility and risk of MetS.

We identified modest increase in the risk of developing DM in ICSI treated men but without reaching the level of statistical significance in any of the analyses we performed. Other studies have demonstrated more or less clearly significantly increased risk of DM in sub-fertile men^28–30^. In one of the biggest cohorts, among 39 000 couples undergoing ART, only the aspermic, azoospermic and severely oligospermic men were found to be with increased risk for DM^28^. The majority of these men might have not been identified in our study since many of them might have not succeed to become biological fathers, adopted children or performed IVF with donated sperm, thus inferring selection bias to our results.

Our analysis established lower risk for filled DM and HT prescriptions in men from the IVF group. These results seem to support our assumption that men with impaired fertility are significantly less prevalent in the IVF group as compared to those ICSI treated. Previous analyses have already shown IVF couples not to be representative for the entire population due to the lower morbidity possibly related to a higher socio-economic status, which may not necessarily be expressed by higher educational level only. Thus, although our finding was robust for adjustment to educational level, other socio-economic variates as income, ethnicity, religion^14,15,31^, could not be accounted for as co-variates using our registry data.

Our study has several strengths as being population-based and having a large size thanks to use of national registers representing men from all socioeconomic backgrounds. The registries provide high quality unbiased (not self-reported) data with high accuracy covering 97–99% of all births and 99% of all drug prescriptions^16,32^. The study has also several limitations. The ICSI group may include men with normal or only slightly subnormal fertility. This would be the case with cancer treated patients where frozen sperm is used, or in cases when the couple had several failed standard IVF attempts which, in clinical practice, leads to switching to ICSI. At the same time sub-fertile men also may be represented in the non IVF/ICSI group. Furthermore, the subjects with most severe infertility, who did not succeed to become fathers, are also excluded. On the other hand, some severe cases of male infertility might have been treated with sperm donation and intrauterine insemination. Since our registry data cannot distinguish them, additional selection bias might be inferred by putting them in our non-IVF/ICSI group. However, all these shortcomings tend to diminish the differences between the three groups of fathers and cannot explain the statistically significant associations found by us. Furthermore, registry data cannot provide causative explanation to our findings in regard to infertility diagnosis, neither can provide data to account for the female factor in the couple.

Men do, generally, to a less degree than women consume health care for disease preventing measures and the subgroup of those with fertility problems represent a significant proportion of the general population. Our analysis showed various chronic diseases to be already more prevalent in men at the time of ICSI procedure but also an increased risk for subsequent prescription of anti-HT drugs. Previous studies^4^ have shown that subclinical and yet undetected metabolic disturbances are present when sub-fertile men are referred for infertility treatment. At the same time the majority of the fathers in our cohort are relatively young and it is plausible that there is a substantial amount of cases which are not yet undergoing medical treatment thus underestimating the prevalence of components of metabolic syndrome in our cohorts. Men undergoing ART might be more likely to be medicalised for a non-previously identified medical condition which, however, does not explain the difference between the ICSI and the IVF men seen in our study. Thus, longer follow-up is needed to provide more information about the magnitude of the increased morbidity rate in those men as well as timing in relation to ART and, thereby options for preventive measures at their contact with the health care system.

## Conclusions

Our analysis revealed statistically significant increase in the risk of developing hypertension and various metabolic disorders requiring prescribed medicines when using ICSI as proxy for male infertility. Whereas our study identifies ICSI-treated men as being at risk for metabolic and cardiovascular disease it cannot answer the question regarding the causal factors driving this association.

## Data Availability

The data that support the findings of this study are available from Swedish National Board of Health and Welfare but restrictions apply to the availability of these data, which were used under license for the current study, and so are not publicly available. Data are however available from the authors upon reasonable request and with permission of Swedish National Board of Health and Welfare.
